# Biphasic Peritoneal Mesothelioma Is a Rare Tumor and a Diagnostic Challenge: A Case Report

**DOI:** 10.7759/cureus.51725

**Published:** 2024-01-05

**Authors:** Eihab A Subahi, Abdalla Fadul, Abdelaziz Mohamed, Ahmed Alsayed, Elrazi A Ali, Sagda Sayed, Salma Mustafa, Bara Wazwaz, Mohamed H Fadul

**Affiliations:** 1 Internal Medicine, Hamad Medical Corporation, Doha, QAT; 2 Internal Medicine, One Brooklyn Health/Interfaith Medical Center, Brooklyn, USA; 3 Psychiatry, Hamad Medical Corporation, Doha, QAT; 4 Pathology, Hamad Medical Corporation, Doha, QAT; 5 Faculty of Medicine, University of Khartoum, Khartoum, SDN

**Keywords:** mesothelioma histopathology, case report, biphasic mesothelioma, peritoneal mesothelioma, malignant mesothelioma

## Abstract

Malignant peritoneal mesothelioma (MPM) is a rare subtype of mesothelioma. There are three main histological subtypes of mesothelioma: epithelioid, sarcomatoid, and biphasic (mixed). Risk factors include asbestos exposure, previous radiation, and some germline mutations. Treatment includes surgical resection of amenable tumors or cytoreductive surgery and hyperthermic intraperitoneal chemotherapy.

We present a 34-year-old male who presented with weight loss, night sweats, and pleuritic chest pain and was found to have ascites with peritoneal nodularity on abdominal imaging. He had a history of tuberculosis contact, but no history of asbestos exposure. After a long challenging and interesting diagnostic process, he was subsequently diagnosed with biphasic MPM. The diagnostic challenge stems from not only the rarity of the tumor but also from the absence of risk factors, the unavailability of some special laboratory investigations, in addition to the potentially misleading effect of tuberculosis exposure history, a top differential diagnosis in the case.

This is a case report of a really challenging and totally unexpected diagnosis of biphasic peritoneal mesothelioma in a patient with tuberculosis exposure, constitutional symptoms, but no history of asbestos exposure. It highlights the diagnostic process as well as the importance of early diagnosis to improve the overall survival of such malignancies.

## Introduction

Mesotheliomas are aggressive tumors that originate from serous mesothelial surfaces, mainly the pleura and the peritoneum [1]. They have three main histological subtypes: epithelioid, sarcomatoid, and biphasic (mixed) mesotheliomas. Malignant peritoneal mesothelioma (MPM) is a rare subtype of mesothelioma that constitutes 10-30% of mesotheliomas, with an incidence of 300 patients per year in the United States [2,3]. Multiple risk factors have been associated with peritoneal mesothelioma, including exposure to asbestos or non-asbestos materials (e.g., mineral fibers), radiotherapy for previous malignancy, chronic inflammatory conditions, and germline mutations (e.g., BRCA1-associated protein 1 (BAP1) inactivation syndrome) [2,4]. The incidence of MPM is higher in males (60%) compared to females (40%), but the overall proportion of mesothelioma in females is higher on the peritoneal side than on the pleural side [5]. Adding to the limited case reports on this rare malignancy, this case report highlights the challenging process of diagnosing such tumors as well as the importance of early diagnosis in limiting patient and family suffering. It also covers the current recommendations for peritoneal mesothelioma diagnosis and the main treatment options.

## Case presentation

A 34-year-old Bangladeshi gentleman was diagnosed with ankylosing spondylitis about six years ago and has been treated with adalimumab and sulfasalazine. He presented in May 2023 with pleuritic chest pain, subjective fever, night sweats, and weight loss for two months (5 kg). Previous symptoms were associated with vague postprandial abdominal pain. He worked as a farmer and has a history of recent contact with a pulmonary tuberculosis (TB) case (roommate). He had no history of asbestos exposure, smoking, or alcohol consumption. Upon assessment in the emergency department, there was no objective fever, and vital signs were within the normal range. Physical examination showed diffuse abdominal tenderness with positive shifting dullness. Chest examination revealed reduced air entry on both lung bases. Basic laboratory investigations were within the normal range except for an elevated C-reactive protein (55.3 mg/L). Chest X-ray revealed right pleural effusion with underlying collapse/consolidation along with atelectatic changes in the bilateral lower zones.

**Figure 1:**
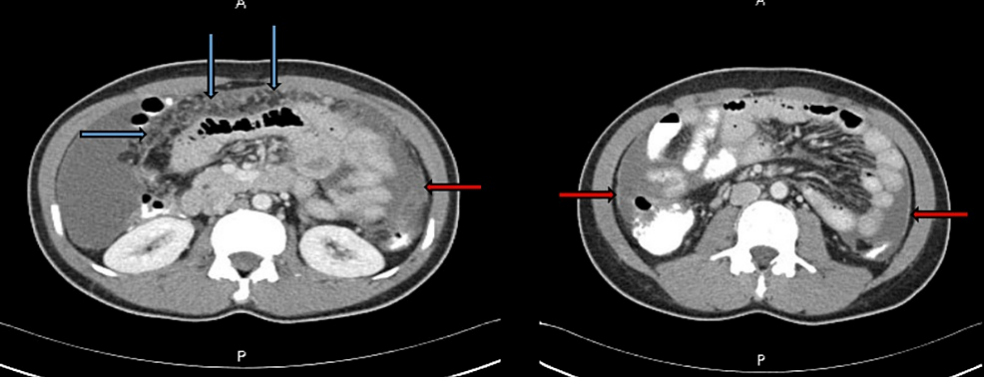
Abdominal CT scan showing diffuse omental thickening and cake (blue arrows) and peritoneal thickening (red arrows).

**Figure 2:**
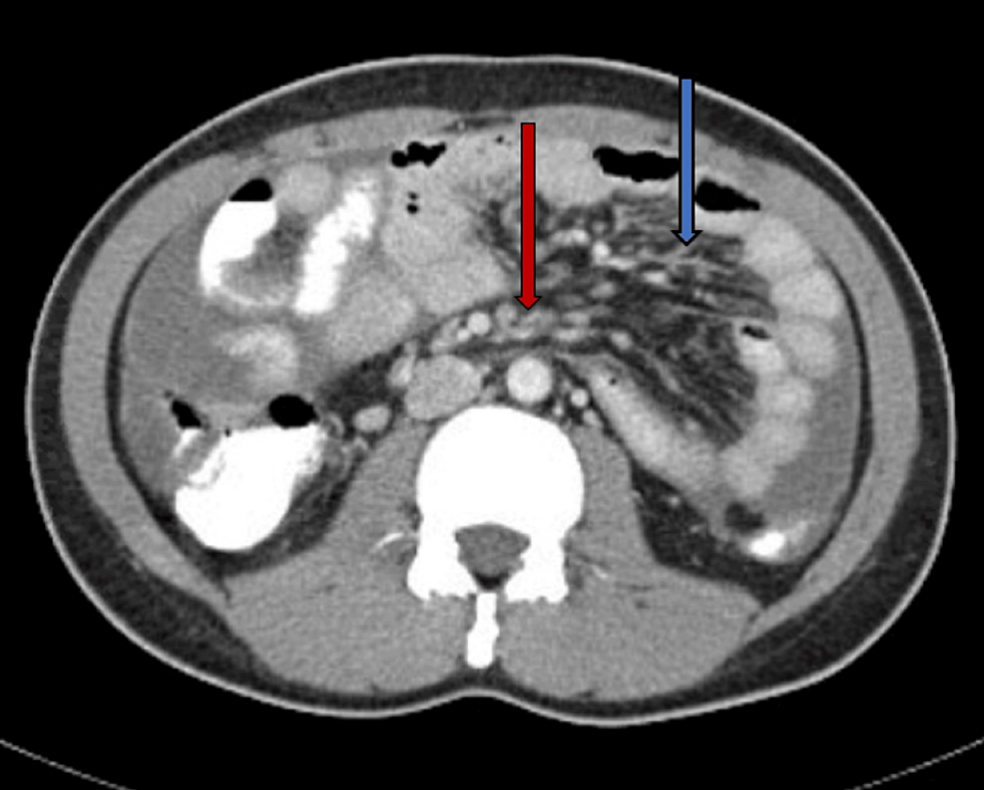
CT scan of the abdomen showing mesenteric thickening (blue arrow) and mesenteric nodularity (red arrow).

He was admitted to the hospital as a suspected TB chest infection and placed under airborne isolation. Work-up for TB including two sputum smears for acid-fast bacilli (AFB) and sputum polymerase chain reaction (PCR) and culture for *Mycobacterium tuberculosis* were all negative. Thoracic computed tomography (CT) scan with contrast showed a right-sided small pleural effusion with adjacent basal lung atelectasis and a small area of focal pleural thickening in the left upper and lower lung lobes, but no definite lung consolidation, cavitary lesions, or tree-in-bud nodules. CT scan of the abdomen and pelvis with contrast showed diffuse peritoneal thickening, edema, and nodularity throughout the omentum and mesentery, moderate ascites, and widely spread moderately enlarged mesenteric lymph nodes with moderate thickening of the mesentery. It also showed two enlarged necrotic right para-rectal lymph nodes (the largest measuring 2.1 cm in the short axis) with enlarged right paracaval lymph nodes with no organomegaly (Figures 1, 2).

The abdominal CT scan findings were reported as highly suspicious of abdominal TB. The patient underwent a diagnostic laparoscopic peritoneal biopsy for suspected peritoneal TB on day eight of admission. Laparoscopy revealed multiple whitish intra-abdominal wall lesions, multiple intra-abdominal adhesions, and ascitic fluid. The patient was discharged on day nine with no post-procedural complications and referred to the post-discharge clinic to follow the ascitic and tissue biopsy reports.

Upon clinic visit after one week, the patient continued to experience his presenting symptoms, including fever and night sweats. The sputum smears for AFB and cultures for *M. tuberculosis* from the ascitic fluid aspiration and peritoneal/abdominal wall biopsies were negative, and ascitic fluid cytology showed no evidence of malignant cells. Abdominal wall biopsy revealed atypical mesothelial proliferation without obvious granulomatous inflammation with an unusual immunostaining profile. Because of the lack of essential stains in the local histopathology lab, the tissue sample was sent for further opinion to an overseas lab that reported a myogenic epithelioid neoplasm of uncertain line of differentiation. Two weeks later, the patient was seen again in the clinic with no reported clinical improvement, and he agreed to repeat the laparoscopic peritoneal biopsy. He was re-admitted, and a CT of the abdomen with contrast was repeated and showed a demonstration of the same findings on the previous CT scan with a suspicion of TB peritonitis. A second diagnostic laparoscopy with multiple peritoneal biopsies and ascites for cytology was performed (five weeks after the first encounter) and went uneventfully; the patient was discharged a day later.

Ascitic fluid analysis showed lymphocytic effusion; repeated smears for AFB as well as PCR and culture for *M. tuberculosis* were negative. Peritoneal biopsies showed atypical epithelioid proliferation (as in the first sample). The immunohistochemical stains for cytokeratin (CK) 7, CK AE1/AE3, cell adhesion molecule (CAM) 5.2, D2-40, WT1, calretinin, and desmin (patchy) were positive in the lesion, while CD34 was negative (all immunostains showed appropriate immunoreactivity). Further immunostains, including thyroid transcription factor 1 (TTF1), epithelial membrane antigen (EMA), MOC31, prostate-specific antigen, NKX3.1, special AT-rich sequence-binding protein 2 (SATB2), and CDX2, were all negative in the lesional cells.

**Figure 3:**
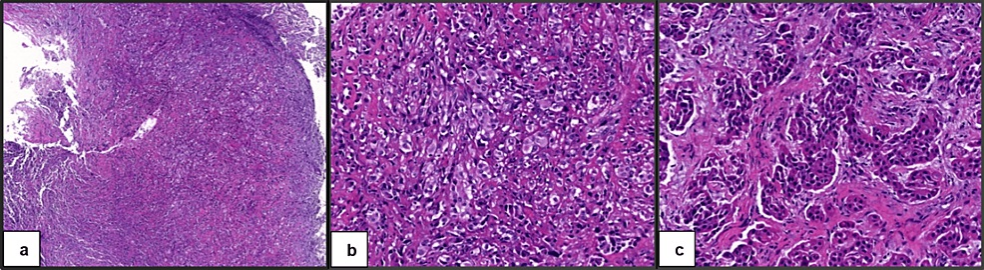
Peritoneal biopsy stained with hematoxylin and eosin (H&E). The characteristic biphasic pattern is shown with 5x magnification (a) and 20x magnification (b); the epithelioid component is shown with 20x magnification (c).

The histopathologist's impression was that “the pattern of infiltration is of a malignant tumor and immune profile is strongly in favor of a mesothelial proliferation over carcinoma. Primary malignant mesothelioma of the peritoneum is rare but the available clinical history fits that. Given the rarity of this tumor, the tissue sample would be referred again to the overseas lab for a second opinion.” Three weeks later (day 72 of the initial presentation), the overseas laboratory reported that the peritoneal biopsies showed biphasic proliferation, which was predominantly epithelioid (approximately 80%) and focally sarcomatoid (approximately 20%). Immunohistochemical stains performed in the referring facility were reviewed, and additional stains, including BAP1, MTAP, MOC31, and claudin-4, were performed. MOC31 and claudin-4 were negative. MTAP showed a loss of staining in the tumor, and BAP1 showed equivocal staining. The overseas laboratory concluded that the previous findings were consistent with biphasic mesothelioma (20% sarcomatoid, 80% epithelioid), as shown in Figure 3.

Given the challenging nature of the case, the biopsy results were shared with a histopathologist who has special expertise in pulmonary pathology, including mesothelioma, and she agreed with the previous interpretation. The patient was informed about the biopsy results and the diagnosis. A fluorodeoxyglucose (FDG)-positron emission tomography (PET) scan was ordered as there was a suspicion of pleural involvement. A multidisciplinary team (MDT) meeting was planned to discuss further management of this case.

## Discussion

MPM is a rare clinical entity, accounting for approximately 10-30% of all mesotheliomas [2,3]. It arises from the mesothelial lining of the peritoneum and spreads extensively within the confines of the abdominal cavity. No imaging modality has proven sensitive or specific for peritoneal mesothelioma diagnosis. CT scan of the abdomen and pelvis with intravenous contrast is initially used, and then a confirmatory CT-guided core needle biopsy or laparoscopic biopsy may both provide sufficient material to make a tissue diagnosis [6]. The pattern of involvement is usually diffuse and widespread involvement of the peritoneal cavity with tumor infiltration and irregular/nodular thickening of the peritoneum in a sheet-like fashion, usually with moderate to extensive ascites. Less commonly, there is a focal pattern of involvement with a dominant intraperitoneal mass with or without associated peritoneal studding.

MPM is classified histologically into three subtypes: epithelial, sarcomatoid, and biphasic (mixed). The epithelioid subtype is the most common, noted in approximately 75% of MPM patients, and has the best prognosis. Approximately 25% of MPM patients have the biphasic type, while the sarcomatoid subtype is exceedingly rare; both of these subtypes have a significantly worse prognosis [7]. Biphasic peritoneal mesothelioma is defined as epithelioid-based, with at least 10% of the composition being sarcomatoid [8]. Our patient was diagnosed with biphasic MPM based on the histological pattern and immunohistochemistry profile, with a shared opinion from the Mayo Clinic.

Malignant mesothelioma is difficult to diagnose histologically. The most recent recommendation is to use at least two mesothelial markers (calretinin, WT1, cytokeratin 5/6, and D2-40 are the most sensitive) and two carcinoma markers with higher than 80% sensitivity and specificity such as MOC31 and BerEp4 monoclonal antibodies, BG8 blood antigen, and carcinoembryonic antigen (CEA). If any of the findings are inconsistent, additional markers will be required [9,10].

Because of the low frequency of disease spread outside of the peritoneum, a staging evaluation for distant metastases is generally not needed, unless there are symptoms suggesting metastases to a distant organ. Because most MPMs are diffuse and difficult to resect, chemotherapy is considered the mainstay of treatment. On the other hand, localized peritoneal mesotheliomas may be treated surgically [3]. Currently, the most commonly used approaches are cytoreductive surgery (CRS) and hyperthermic intraperitoneal chemotherapy (HIPEC) for diffuse MPM, with a significant reduction in morbidity and mortality rates achieved [10,11].

Sarcomatoid-predominant biphasic peritoneal metastases are rapidly progressing and deeply invasive with survival measured in months. Sometimes, the standard treatment of CRS and HIPEC alone is not recommended, as the sarcomatoid variant is so aggressive [12]. Several studies have been carried out, and the first successful case documented combining CRS with a monoclonal antibody that targets the programmed death (PD-1) receptor to obtain a favorable long-term outcome. Nivolumab, a monoclonal antibody that targets the PD-1 cluster of differentiation on suppressor T cell surface, was used in this study. When PD-1 binds its ligands on the T-cell surface, suppressor T-cell lymphocyte function is reduced. By preventing PD-1 from interacting with its ligands, nivolumab stimulates the immune system to initiate its anticancer activity. Long-term survival with sarcomatoid-predominant peritoneal mesothelioma has been reported in the study [12,13].

For patients who are not candidates for CRS/HIPEC due to unresectable disease or medical comorbidities, systemic chemotherapy with contemporary pemetrexed-based regimens achieves response rates comparable to those seen in patients with pleural mesothelioma and is now commonly incorporated into some peritoneal mesothelioma treatments.

A retrospective analysis of the Peritoneal Surface Oncology Group International (PSOGI) registry including data from 33 centers was performed. Survival was reviewed based on mesothelioma type, completion of cytoreduction, and volume of disease. They concluded that long-term survival is achievable in patients with low-volume biphasic mesothelioma after complete macroscopic cytoreduction [14].

## Conclusions

This case report highlights the challenging and totally unexpected diagnosis of biphasic peritoneal mesothelioma in a male patient with TB exposure, ascites, and constitutional symptoms but no history of asbestos exposure. The diagnostic challenge stems from not only the rarity of the tumor but also from the absence of risk factors, the unavailability of some special laboratory investigations, in addition to the potentially misleading effect of TB exposure history, a top differential diagnosis in the case. Most patients have an advanced disease upon diagnosis due to the vague and nonspecific clinical presentation. Therefore, a high index of suspicion is necessary for early diagnosis, which may improve the survival rates and overall prognosis. Our case report also explores the current state of care in addition to the new developments in the field of MPM.
